# How It Will Affect the Worker

**Published:** 1911-06-03

**Authors:** Thos. A. Cook

**Affiliations:** Chairman of the General Committee of Management, West Ham and Eastern General Hospital.


### STATE INSURANCE.

### HOW IT WILL AFFECT THE WORKER.

By THOS. A. COOK, Chairman of the General Committee of Management, West Ham and Eastern General Hospital.

I read with great interest, in the issue of May 27, the article under the above heading by Mr. Walter G. Carnt. There are two or three points that strike me.

For some years I have worked on the Committee of the West Ham and Eastern General Hospital. This hospital is, perhaps, in a typically poor district, as West Ham has become the sleeping place of London's workers; practically none of the manufacturers reside in the neighbourhood, and there are very few residents who may be called even comfortably off. Mr. Carnt states: "Legislation of recent years has made the struggle rather more arduous, but no previous legislation has created in the minds of hospital administrators the dismay that is taking possession of them the more the provisions of the Insurance Bill are studied. The backbone of the income of most of the voluntary hospitals is the annual subscription list."

Whether it has been due to legislation, or whether it has been due to the enhanced cost of living which has been in vogue of recent years, or not, it is difficult to say; but certainly in respect to the West Ham Hospital, annual subscriptions have been increasingly difficult to obtain. Last year with a total expenditure of £6,761 only £1,159 was due to annual subscriptions. This was a falling off from 1909 and a still further falling off from 1907-1908.

The annual subscriptions during last year were insufficient to pay for the total expended on either "maintenance," or "domestic expenses," or "salaries and wages": the total expended on either of these items was considerably more than the total of annual subscriptions. Donations, including those at the annual dinner (which was a rather successful function last year) amounted only to £2,378 19*s.* 9*d.*; whereas in the year 1862 the donations to the South Essex Dispensary (which was the seed from which the hospital has sprung) amounted to £2,843 14*s.* from eighty donors. Thus you will see how hard a struggle it is for a voluntary hospital in the East of London to make ends meet. If it had not been for the support from King Edward's Hospital Fund and other generous organisations during the past years, I shudder to think what we should have done.

I agree with Mr. Carnt most emphatically in thinking that the work of hospitals, especially in the *in*-patient department, will be little relieved. Considering that in the aforementioned hospital any extension of accommodation leads to an immediate extension of demand (showing that the neighbourhood is not over-supplied with hospital accommodation—in fact, is very much under-supplied at the present moment) I cannot look forward to any lessening of the demand on the *out*-patient department.

I have always set my mind against State control of hospitals, because I believe that voluntary efforts, such as are put forward in the support of West Ham Hospital, do a very great deal more good for very much less money than would be the case under State control. The Committee give their services voluntarily and put in a large number of hours of disinterested work. The honorary medical staff give their services voluntarily, and the same may be said with regard to their work; and the officials probably accept much more reasonable salaries than would be given them were they State servants.

But I heartily endorse Mr. Carnt's suggestion that the State might contribute to the voluntary hospitals, leaving their control in the hands of the present authorities, particularly seeing what wonderfully efficient extra control the inspection and report of the King's Fund have created.

I think that it is most urgent that a meeting in London to consider the Bill from the voluntary hospital point of view should be convened, and would suggest that perhaps the secretaries of King Edward's Hospital Fund might take this upon themselves. They have called meetings to consider various points which have been well attended by the chairmen and secretaries of the London hospitals, but I think the scope of this meeting should go further afield and extend to the provinces.

Mr. Richard J. Cole, of Addenbrooke Hospital, seems to share with me the view that the work of the voluntary hospital in the provinces will be decreased very little, if at all, should the Bill become law. It is a well-known fact that the out-patients of most of the voluntary hospitals are very largely composed of the wives and children of the workers, and the workers seldom attend except in the case of accidents.

In the case of West Ham, the casualties admitted are in very large proportion to the total number of out-patients. Last year, out of 28,980 total out-patients, attending 131,000 times, 14,278 were casualties attending 42,294 times. It is hardly probable that the effect of the Bill will be to reduce the total number of casualties.

